# Heterogeneity in breeding productivity is driven largely by factors affecting nestlings and young fledglings in an imperiled migratory passerine

**DOI:** 10.1002/ece3.11327

**Published:** 2024-05-20

**Authors:** Darin J. McNeil, Amanda D. Rodewald, Viviana Ruiz‐Gutierrez, Cameron J. Fiss, Jeffery L. Larkin

**Affiliations:** ^1^ Department of Forestry and Natural Resources University of Kentucky Lexington Kentucky USA; ^2^ Cornell Laboratory of Ornithology Ithaca New York USA; ^3^ Department of Natural Resources and the Environment Cornell University Ithaca New York USA; ^4^ Department of Biological Sciences University of Pittsburgh Pittsburgh Pennsylvania USA; ^5^ Department of Biology Indiana University of Pennsylvania Indiana Pennsylvania USA; ^6^ American Bird Conservancy The Plains Virginia USA

**Keywords:** fledgling survival, habitat conservation, migratory songbirds, nest survival, productivity

## Abstract

Identifying factors that drive variation in vital rates among populations is a prerequisite to understanding a species' population biology and, ultimately, to developing effective conservation strategies. This is especially true for imperiled species like the golden‐winged warbler (*Vermivora chrysoptera*) that exhibit strong spatial heterogeneity in demography and responds variably to conservation interventions. Habitat management actions recommended for breeding grounds conservation include timber harvest, shrub shearing, and prescribed fire that maintain or create early successional woody communities. Herein, we assessed variation in the survival of nests [*n* = 145] and fledglings [*n* = 134] at 17 regenerating timber harvest sites within two isolated populations in Pennsylvania that differed in productivity and response to habitat management. Although the overall survival of nests and fledglings was higher in the eastern population than the central population, this was only true when the nest phases and fledgling phases were considered wholly. Indeed, survival rates of nestlings and recently fledged young (1–5 days post‐fledging) were lower in the central population, whereas eggs and older fledglings (6–30 days post‐fledging) survived at comparable rates in both populations. Fledglings in the central population were smaller (10% lower weight) and begged twice as much as those in the eastern population, suggesting food limitation may contribute to lower survival rates. Fledgling survival in the central population, but not the eastern, also was a function of habitat features (understory vegetation density [positive] and distance to mature forest [negative]) and individual factors (begging effort [negative]). Our findings illustrate how identifying how survival varies across specific life stages can elucidate potential underlying demographic drivers, such as food resources in this case. In this way, our work underscores the importance of studying and decomposing stage‐specific demography in species of conservation concern.

## Graphical abstract

Appalachian golden‐winged warbler declines have been linked to breeding productivity, however, the factors that drive disparities in breeding output remain unknown. In two regions of Pennsylvania, the survival of eggs and older fledglings was similar, but nestlings and young fledglings were different. These differences may be related to prey availability.

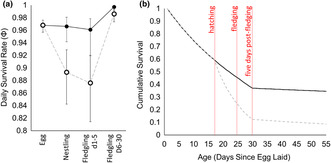

## 1 INTRODUCTION

Understanding the factors that drive variation in vital rates is central to many aspects of population biology (Arcese et al., 1992; Gallardo et al., 2016; Jones et al., 2020) and community ecology (Fortin et al., 2005; Pace et al., 1999). Although vital rates are widely recognized to vary across ages or life stages, many studies apply only a coarse lens that obscures finer‐scale variation that may have profound implications for population persistence and adaptation (Cortázar‐Chinarro et al., 2017; Milner et al., 2007). For example, low recruitment into a population may be caused by low survival of eggs, nestlings, young fledglings, or juveniles—each of which may be affected by different factors (Bilde et al., 2007; Creel & Creel, 2015; Naef‐Daenzer & Grüebler, 2016). Assessments of relative habitat quality that do not break down ecological needs into appropriate demographic phases may draw incorrect conclusions about the full lifecycle needs of focal species (Allen et al., 2022; Mills et al., 1999). In this way, failure to recognize such fine‐scale variation may result in management interventions being directed to ages or life stages that are not limiting (Petrovan & Schmidt, 2019; Saether et al., 1999).

There are many ways that researchers can decompose coarse vital rates (i.e., birth, death, immigration, and emigration; Hanski & Gilpin, 1991; Pulliam, 1988), into smaller components (Clutton‐Brock & Sheldon, 2010; Heppell et al., 2000; Sæther & Bakke, 2000; Silvertown et al., 1993). For example, adult survival can be modeled as a function of life cycle stage (e.g., Norris & Marra, 2007), sex (Nichols et al., 2004), or other life‐history components (Menges, 1992). Decomposing vital rates into subcomponents in this way and exploring how factors may differentially affect each can prove fundamental to both understanding population ecology and conserving species of concern (Faaborg et al., 2010; Greenberg & Marra, 2005). Indeed, ecologists have long recognized the importance of stage‐specific demography (Johnson, 1979; Mayfield, 1975; Rotella et al., 2004) but most studies focus on a single life stage (e.g., Aldinger et al., 2015; Richardson et al., 2020) or fail to consider variation within a stage (e.g., Confer et al., 2010; McNeil et al., 2017). Such failure can prove problematic when critical variation is attributed to broad life stages when it can be more appropriately attributed to a more precise phase of the lifecycle. For instance, in many species, survival during early life stages (i.e., immatures) is among the most important drivers of population growth (Clark & Martin, 2007; Radchuk et al., 2013; Vonesh & de la Cruz, 2002) but meaningful variation can be found at even finer‐scales, such as embryos, larvae, nestlings, fledglings, independent juveniles, etc. (Meier et al., 2010; Radchuk et al., 2013).

In conservation, understanding fine‐scale demographic variation and the causes of heterogeneity in a species' vital rates enhances knowledge of the factors that limit population growth (Bookhout, 1994; Primack, 2006). Identifying the factors that limit productivity for rare or endangered species may allow biologists to develop strategies to mitigate these factors in ways that enhance vital rates and, thus, conservation outcomes (Begon & Townsend, 2021). This is especially true if the factors that affect vital rates are those that can be manipulated by management (Bookhout, 1994). For example, early studies on the Kirtland's warbler (*Setophaga kirtlandii*) indicated that population growth was limited by low reproductive output—especially due to the effects of brood parasitism by the brown‐headed cowbird (*Molothrus ater*; Mayfield, 1961, 1972). In the 1970s, the United States Fish and Wildlife Service began management efforts, informed nest survival studies (e.g., Mayfield, 1961, 1972), focused on trapping and removing cowbirds around Kirtland's warbler breeding sites (Brown et al., 2017). These management efforts, coupled with ambitious habitat restoration efforts, drastically reduced parasitism rates, increased reproductive output, and, ultimately, allowed the species to recover (Brown et al., 2017; Cooper et al., 2019). Understanding the factors that drive variation in vital rates is of particular interest when some populations flourish while others decline (Bookhout, 1994; Willi & Hoffmann, 2009). In cases of strong intrapopulation heterogeneity in vital rates, identifying the factors that drive vital rates in different populations can help elucidate targets for potential management.

Another imperiled songbird whose responses to conservation are driven by breeding productivity is the golden‐winged warbler (*Vermivora chrysoptera*; Confer et al., 2020; McNeil, Rodewald, Robinson, et al., 2020). This Nearctic‐Neotropical migrant songbird breeds across two sub‐populations: the Great Lakes and the Appalachian Mountains regions (Confer et al., 2020; Fink et al., 2022). Overall, the species has been experiencing sustained and dramatic declines at −1.85% per year since at least 1966 (Sauer et al., 2020) or perhaps longer (Hill & Hagan, 1991). These declines are driven by a wide variety of factors (e.g., cowbird parasitism, wintering grounds losses, etc.), however, the most significant driver is the loss of early‐successional nesting habitat (Roth et al., 2019). At broad extents, golden‐winged warblers exhibit strong heterogeneity in population trends among regions with states in the “Great Lakes” sub‐population declining slower (e.g., Minnesota: stable, Wisconsin: −2.39%/year, Michigan: −4.15%/year) than the “Appalachian Mountains” sub‐population (e.g., Pennsylvania: −6.89%/year, West Virginia: −6.66%/year).

Breeding grounds management recommendations describe restoration practices that hold promise for restoring habitat (e.g., timber harvest, shrub management, etc.; Bakermans et al., 2015; McNeil, Rodewald, Robinson, et al., 2020; Roth et al., 2014, 2019), but restoration efforts have yet to produce enough habitat to recover populations (Litvaitis et al., 2021). Moreover, even within a single region, local populations can vary widely in demographic characteristics (Aldinger, 2018; McNeil, Rodewald, Robinson, et al., 2020). For example, golden‐winged warblers in eastern Pennsylvania experienced high breeding output (3.07 juveniles/pair/year), while those in central Pennsylvania generated low breeding productivity rates (1.08 juveniles/pair/year; McNeil, Rodewald, Robinson, et al., 2020). Similar patterns have been reported in other portions of the species' range (e.g., West Virginia) whereby local population breeding output drives their differential responses to conservation action (Aldinger, 2018). Although variation in breeding productivity is known to affect the population dynamics of golden‐winged warblers in Central Appalachian (McNeil, Rodewald, Robinson, et al., 2020; McNeil, Rodewald, Ruiz‐Gutierrez, et al., 2020), the degree to which specific life stages or ecological factors contribute to patterns remains poorly understood. We hypothesized that factors driving fledgling survival in restored warbler habitat would be responsible for breeding output differences because fledgling habitat was not an explicit consideration in the development of the species' best management practices (Roth et al., 2019). To test this hypothesis and identify ecological factors and life‐history stages responsible for differential reproductive rates in restored habitats in this system, we assessed variation among key components of breeding productivity in eastern Pennsylvania (high breeding productivity) and central Pennsylvania (low breeding productivity). Specifically, we quantified (1) variation in survival rates across and within key life stages (egg, nestling, and fledgling), (2) effects of breeding phenology and micro‐habitat on daily nest survival rate, and (3) the influence of individual, phenological, micro‐habitat, and stand‐scale variables on fledgling survival. We sampled recently created overstory removal timber harvests that conformed to species‐specific breeding habitat management guidelines (described below).

## 2 MATERIALS AND METHODS

### 2.1 Study area

We selected multiple replicate study sites within two of the Appalachian Mountains' sub‐population's densest breeding “local populations” of golden‐winged warblers (Fink et al., 2022; Wilson et al., 2012): eastern Pennsylvania (2014–15) and central Pennsylvania (2016–17). Although our two local populations were studied in different years, weather conditions sampled in each region were comparable (i.e., one drought year and one nondrought year in each; Appendix 1). Within both local populations, we monitored demography in habitats treated using golden‐winged warbler best management practices (Bakermans et al., 2011; Roth et al., 2019). Specifically, study sites were deciduous overstory removal timber harvests (5–10 years post‐harvest) leaving 2.2–8.9 m^2^/ha of residual basal area (Roth et al., 2019). All habitats occurred at high‐elevations (300–750 m.a.s.l.) and within heavily forested landscapes (>80% forest cover).

### 2.2 Eastern Pennsylvania

Our eastern Pennsylvania local population was within the Glaciated Low Plateau section of the Appalachian Plateaus Physiographic province and characterized by moderate elevation (395–550 m.a.s.l.) rolling hills punctuated by abundant wetlands (Cuff, 1989; Shultz, 1999; White & Chance, 1882). This region is dominated by mature forests of mixed coniferous‐deciduous and deciduous composition, with northern hardwood and mixed‐oak (*Quercus* spp.) communities most common (McCaskill et al., 2009). Golden‐winged warblers nest within two habitat types in the eastern Pennsylvania local population: natural wetlands and managed early‐successional forest (McNeil et al., 2018). Within eastern Pennsylvania, we focused our survey efforts on Delaware State Forest (WGS84 decimal degrees: 41.2338*°* latitude, −75.0739*°* longitude), which includes 33,000 ha of publicly owned forest in Pike, Monroe, Northampton, and Carbon Counties, though we only worked in the former two counties. Portions of Delaware State Forest are harvested on a rotational basis with the goal of diversifying forest age classes. In the eastern Pennsylvania local population, we randomly selected six regenerating timber harvests ranging in size from 7 to 68 ha meeting the best management criteria described above.

### 2.3 Central Pennsylvania

Our central Pennsylvania local population was within the Deep Valley section of the Appalachian Plateaus Physiographic province, which is characterized by high‐elevation ridges (366–700 m.a.s.l.) and deep, narrow, steep‐sloped valleys. This landscape is dominated by mature forests with mixed‐ and deciduous (e.g., northern hardwood, mixed‐oak) forest types most common (McCaskill et al., 2009). Unlike in eastern Pennsylvania, wetlands were rare in central Pennsylvania (Cuff, 1989; Fry et al., 2011) and golden‐winged warblers were therefore restricted to upland habitats in this local population (Fiss et al., 2020, 2021). In central Pennsylvania, we surveyed Sproul State Forest (WGS84 decimal degrees: 41.1880*°* latitude, −77.8785*°* longitude) and Pennsylvania State Game Lands 100 (SGL 100; WGS84 decimal degrees: 41.0930*°* North, −78.0098*°* East), both of which are managed to diversify forest age classes for the benefit of forest‐ and wildlife health. Sproul State Forest and SGL 100 occur across a collective 194,000 ha of forest land in Centre and Clinton Counties. We randomly selected 11 timber harvests (18–262 ha in size) that met golden‐winged warbler best management practices after removing from consideration those sites where golden‐winged warblers were absent or at very low densities. Managed sites in central Pennsylvania ranged from 18 to 262 ha in size. Not only do geomorphology and land cover composition differ between the landscapes, but golden‐winged warbler full‐season productivity contrasts sharply as well: 3.07 juveniles/pair/year (95% CI: 2.62–3.53) in the eastern Pennsylvania local population versus 1.08 (95% CI: 0.80–1.37) in the central Pennsylvania local population (McNeil, Rodewald, Robinson, et al., 2020). Data from 2018 revealed that the proportion of adults in each population that were first‐time breeders (i.e., “second year”, [SY]) was comparable: eastern Pennsylvania's population is 22% SY (*n* = 50) versus 29% SY (*n* = 62) in central Pennsylvania (E. Keele, unpublished data).

### 2.4 Nest searching and monitoring

Following the methods of McNeil et al. (2017), we located nests using a combination of systematic sampling and opportunistic observation of adult behavior. Systematic sampling consisted of a trained field technician hiking through habitats and physically searching through all vegetation within which nests could conceivably be placed (Confer et al., 2020). Observations of adult behaviors involved attempting to locate birds nesting in all portions of each site and following adults to their nests when cues were presented (e.g., alarm calls, etc.). We monitored nests every 2–3 days, more frequently as fledging approached (Martin & Geupel, 1993). For analytical purposes, nests were considered “initiated,” at the earliest, once they contained at least one egg (i.e., nests without eggs were not considered) and nests were considered “successful” if at least one chick fledged (Martin & Geupel, 1993).

### 2.5 Fledgling telemetry

To monitor fledgling survival, we marked nestling golden‐winged warblers either (1) immediately prior to fledging (7 days old) or (2) on the day of fledging (9 days old). See Appendix 2 for aging criteria. From each brood, 1–2 nestlings were randomly selected from each nest (mean clutch size = 4.7 in our study areas) for measurement, banding (a USGS aluminum band and a single plastic color band), and transmitter attachment. To obtain weight data, each bird was placed in a small plastic bottle and weighed using a digital scale (0.01 g accuracy). Transmitters were attached using a figure‐eight harness (Rappole & Tipton, 1991) secured over the synsacrum using <1 mm elastic cord. The combined mass of the transmitter, glue, and harness was 0.39 g: <5% of the mean mass of a fledgling (Fair et al., 2023). Processing for each fledgling was approximately 2–3 min. Radio transmitters used in our study (Blackburn Transmitters Inc., Nacogdoches, TX) had an expected battery life of about 30 days. After transmitters were attached, each chick was returned to the initial capture location (i.e., perch/nest). We tracked fledglings daily, until either mortality or transmitter failure, using the homing method, a Yagi H‐type antenna, and a hand‐held radio receiver. At each fledgling location, we identified geographic coordinates using a handheld GPS unit, conducted a vegetation survey (see Micro‐habitat quantification section, below), and estimated begging rates (i.e., percent of time a fledgling vocalized during our ~5‐min observation).

### 2.6 Micro‐habitat quantification

At nest locations, we employed the nest vegetation sampling protocol developed by the Golden‐winged Warbler Working Group (Aldinger et al., 2015; McNeil et al., 2017). This protocol required us to (1) estimate percent cover of woody vegetation, *Rubus* spp., vines, forbs, grass, leaf litter, and bare ground within 1‐m of the nest, (2) count shrubs in three height classes (0.5–1 m, 1–2 m, and >2 m) within 5 m, (3) estimate the average height of shrubs and saplings as well as tally and measure diameter‐at‐breast‐height (DBH) of all trees and snags within 11.3‐m, and (4) measure the presence/absence of grass, forb and *Rubus* spp. cover at 2.26‐m intervals along four 11.3‐m transects in each cardinal direction using an ocular tube (James & Shugart, 1970).

Within a 1‐m radius of each fledgling location, we visually estimated percent cover of woody vegetation, *Rubus* spp., vines, forbs, grass, leaf litter, and bare ground. We combined *Rubus* spp. and woody into a “nonherbaceous vegetation” class. Vines, forbs, and grass were combined into a “herbaceous” class, whereas leaf litter and bare ground were combined into an “unvegetated” class. We also measured “vertical vegetation cover” at each fledgling location by reading a spherical densiometer in each cardinal direction centered at fledgling locations, held at 1‐m in height (hereafter, “percent vertical vegetation cover”). We recorded “lateral vegetation density” using a density board (Nudds, 1977) read from a 5‐m distance and 1‐m from the ground (% squares >50% covered; see Fiss et al., 2020, 2021). Finally, we measured the basal area at each fledgling location using a 10‐factor basal area prism.

### 2.7 Forest stand quantification

To assess the influence of stand structure on fledgling survival, we used forest inventory data for Delaware State Forest, Sproul State Forest, and State Game Lands 100. Data included maps provided by regional foresters with the following categories: (i) early‐successional (<20 years post‐harvest), (ii) sapling (>50% stocked by trees <15 cm in DBH), (iii) thinned (<50% stocked by trees >15 cm in DBH), (iv) mature (>50% stocked by trees >15 cm DBH), (v) swamp (palustrine stands >50% stocked by trees >15 cm DBH), and (vi) shrubland (palustrine or upland communities <50% stocked by trees and dominated by shrubs). Using these forest inventory data, we analyzed (1) percent cover and (2) proximity (e.g., minimum distance to‐) for each fledgling/day with respect to each cover type. We calculated percent cover using extract by mask in ArcGIS 10.2 (ESRI, 2011) within fledgling home ranges within 1–5 days of leaving the nest, which is when nearly all mortality occurs in the eastern Pennsylvania study site (Jones et al., 2020). Stand‐scale habitat was summarized within 150‐m‐r buffers around each fledgling home range centroid (Vitz & Rodewald, 2010). Because fledgling survival varied over the entire 30‐day post‐fledging period in central Pennsylvania, home ranges for fledglings in this landscape were based on either a 150 m radius buffer (using each bird's centroid location from days 1 to 30) or a minimum convex polygon around all observed locations, using whichever area was larger. Several covariates were too uncommon to allow parameter estimation and were discarded when this occurred: percent sapling/thinned stand (too uncommon in both landscapes), distance to nearest early successional stand (almost always 0, both landscapes), and “percent swamp,” “distance to nearest swamp,” and “distance to nearest sapling stand” covariates were only usable for our eastern Pennsylvania analyses (too uncommon in central Pennsylvania).

### 2.8 Analyses

#### 2.8.1 Nest survival

We used an information theoretic approach (Burnham & Anderson, 2002) to assess factors associated with nest survival. For our “primary” nest survival analyses (in contrast to our “egg survival” and “nestling survival” components, discussed below), we monitored nests from the time we first observed eggs/nestlings until completion (i.e., failure or fledging). We specified logistic exposure models with the “Nest Survival” interface in program MARK (ver.7.1; Colorado State University, Fort Collins, Colorado, US; Dinsmore & Dinsmore, 2007; Rotella et al., 2004). Models were compared with Akaike's Information Criterion adjusted for small sample size (AICc; Akaike, 1973) with those within 2.0 ΔAICc considered to be equally supported (Burnham & Anderson, 2002). The “daily survival rate” (DSR) for each nest was estimated separately for each landscape for the following: (1) *β*_1_ (vegetation covariate), (2) *β*_1_ (ordinal date) + *β*_2_ (vegetation covariate), and (3) *β*_1_ (ordinal date) + *β*_2_ (ordinal date^2^) + *β*_3_ (vegetation covariate). Prior to analysis, we screened data for highly correlated variables (*r* > .7; Sokal & Rohlf, 1969) and ensured that highly correlated variables never appeared in the same model together. We pooled nests from each region together for analyses. In addition to our nest DSR models, we predicted mean “egg stage” survival and “nestling stage” DSR for each landscape using intercept‐only nest survival models for respective stages. A nest “entered” the egg stage when it had ≥1 egg and was successful when ≥1 egg hatched. Likewise, nests entered the nestling stage when they contained ≥1 nestling and were successful when ≥1 nestling fledged.

#### 2.8.2 Fledgling survival

As with nests, we modeled the effects of vegetation covariates on fledgling DSR using an information theoretic approach implemented in Program MARK (“Known Fate” interface; White & Burnham, 1999). We tested combinations of temporal patterns (i.e., fledgling age) with 0–1 vegetation covariates using identical model selection criteria used in nest survival analyses, above. Specifically, we tested (1) *β*_1_ (vegetation covariate), (2) *β*_1_ (fledgling age) + *β*_2_ (vegetation covariate), and (3) *β*_1_ (fledgling age) + *β*_2_ (fledgling age^2^) + *β*_3_ (vegetation covariate). We assessed a variety of patterns of fledgling age on survival because the first few days post‐fledging are the most dangerous in many species (Cox et al., 2014; Naef‐Daenzer & Grüebler, 2016), but the most appropriate pattern was unknown in our system. Prior to analyses, we noticed a distinct pattern of early fledgling mortality (days 0–11 post‐fledging) and constant survival thereafter in eastern but not central Pennsylvania. We, therefore, modeled a quadratic relationship with age in central Pennsylvania and modeled an early quadratic (days 0–11 post‐fledging) + constant survival thereafter (days 12–30) in eastern Pennsylvania. Incorporating the aforementioned temporal predictors, we tested all possible combinations of 0–1 “individual‐level” covariates on fledgling survival: fledge date, mass at banding, daily begging effort, daily movement distance, and year. We repeated this process for microhabitat covariates (e.g., % cover variables, lateral/vertical vegetation density, etc.), and stand‐scale covariates (e.g., distance to nearest mature stand, percent shrubland, etc.). Percent early successional forest was negatively correlated with percent mature forest (*R* > .7) and was not analyzed. Fledglings from each region were pooled together for analyses.

## 3 RESULTS

### 3.1 Nests and fledglings monitored

We monitored the survival of 77 nests in eastern Pennsylvania, the highly productive local population and 79 in central Pennsylvania, the local population with low productivity. In 2017, an unusual, localized hailstorm in central Pennsylvania resulted in the complete failure of nests at one site (*n* = 11 nests; Fiss et al., 2019) so we censored those nests. Of the remaining nests, 46 out of 77 (60%) and 23 out of 68 (34%) successfully fledged at least one young in eastern Pennsylvania and central Pennsylvania, respectively. Nest initiation occurred 7 days earlier (ordinal date 134) in eastern Pennsylvania than in central Pennsylvania (141; Appendix 3). In addition to nests, we tracked the survival of 63 fledglings in eastern Pennsylvania and 60 in central Pennsylvania (total = 123). An additional seven and four fledglings, respectively, were captured (by 30 mm mist net) >5 days post‐fledging (aged via plumage characteristics; McNeil, 2019; *n* = 11 total “older” fledglings) for a final total of 134 fledglings used in our estimates of DSR in the latter period of the post‐fledging period (days 6–30 but not included in our assessments of “young” [days 1–5] fledglings). Of the 123 fledglings monitored from the time of fledging, 73% (46 of 63) survived to independence in eastern Pennsylvania and 41% (34 of 60) survived in central Pennsylvania.

### 3.2 Regional estimates of reproductive vital rates

**FIGURE 1:**
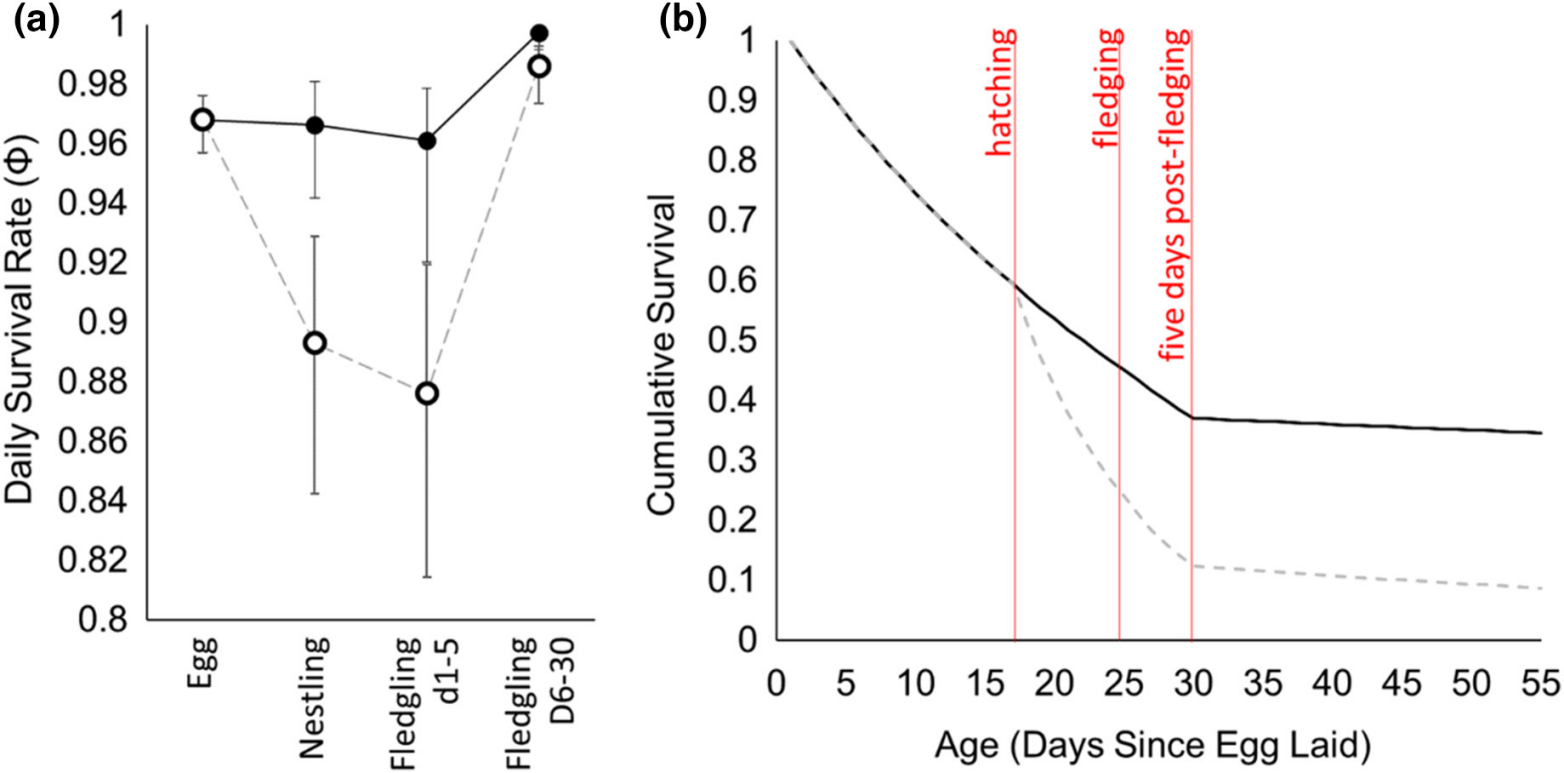
Daily survival rates for golden‐winged warbler (*Vermivora chrysoptera*) life stages from eggs, nestlings, young fledglings (1–5 days post‐fledging) and older fledglings (6–30 days post‐fledging). We modeled our two landscapes, eastern Pennsylvania (solid circles) and central Pennsylvania (open circles) separately. Point estimates are shown along with 95% confidence intervals (error bars; a). Also shown (b) are cumulative survival probabilities across the 55‐day period of parental care exhibited by the species for eastern Pennsylvania (solid) and central Pennsylvania (dashed; b). Predictions were made using the top‐ranked model in respective model sets (see Table 1).

Our models indicated that nests during the “egg phase” experienced similar DSR in both eastern Pennsylvania and central Pennsylvania (DSR: 0.97, 95%CI: 0.96–0.98; Table 1; Appendix 4; Figure 1a). In contrast to egg phase survival, nestling phase survival differed, regionally (Table 1) with eastern Pennsylvania nest DSR remaining high (0.97, 95%CI: 0.94–0.98; Figure 1) but nestling DSR in central Pennsylvania dropped substantially after nestlings had hatched (DSR: 0.89, 95%CI: 0.84–0.93 Figure 1). This disparity continued over the early post‐fledging period (days 1–5; Table 1) where mean DSR remained high in eastern Pennsylvania (DSR: 0.96, 95%CI: 0.92–0.98) and low in central Pennsylvania (DSR: 0.88, 95%CI: 0.81–0.92; Figure 1a). Finally, during the latter portion of the post‐fledging period (days 6–30), both regions supported relatively high DSR (eastern Pennsylvania: 1.00, 95%CI: 0.99–1.00; central Pennsylvania: 0.99, 95%CI: 0.97–0.99; Figure 1). Calculations of cumulative survival demonstrated that warbler survival declined steadily and equally for both regions during the egg stage, however, DSR disparities between began at the time of hatching and continued until young had survived the post‐fledging period for 5 days (Figure 1b). By the time young survived to 5 days post‐fledging, survival probability in eastern Pennsylvania was three times higher (0.37) than in central Pennsylvania (0.12; Figure 1b).

**TABLE 1:** Models explaining survival of golden‐winged warbler (*Vermivora chrysoptera*) life phases in eastern Pennsylvania and central Pennsylvania regions.

Model name	*k*	ΔAICc	*w*	Mod lik.	*β* coefficients
Egg‐phase survival					
Φ (int)	1	0.00	0.59	1.00	—
Φ (int + region)	2	0.76	0.41	0.68	*β*_1_: *−0.35*
Nestling‐phase survival					
Φ (int + region)	2	0.00	0.99	1.00	*β*_1_: **−1.23**
Φ (int)	1	9.66	0.01	0.01	—
Young (1–5 days Fledgling‐phase survival)					
Φ (int + d_2–5_ + region_1_ + region_2–5_)	4	0.00	0.51	1.00	*β*_1_: *0.81*, *β*_2_: **4.52**, *β*_3_: **−2.50**
Φ (int + d_2–5_ + region_2–5_)	3	0.14	0.48	0.93	*β*_1_: **4.52**, *β*_2_: **−2.50**
Φ (int + d_3–5_ + region_3–5_)	3	11.06	0.00	0.00	—
Φ (int + d_3–5_ + region_1–2_ + region_3–5_)	4	13.00	0.00	0.00	—
Φ (int + age + region)	3	17.19	0.00	0.00	—
Φ (int)	1	23.86	0.00	0.00	—
Old (6–30 days Fledgling‐phase survival)					
Φ (int + region)	2	0.00	0.93	1.00	—
Φ (int)	1	5.27	0.07	0.07	—

*Note*: Shown are the number of model parameters (*k*), Δ Akaike's Information Criterion adjusted for small sample size (AICc), AICc weight (*w*) and model likelihood (Mod lik.) for candidate nest survival models. For competing models (ΔAICc <2), model *β* coefficients are shown where *β*_0_ (not shown) represents the intercept and *β*_1_ … *β*_k_ represents model covariate slopes. *β* coefficients are presented in bold if their *β* 95% confidence intervals that do not include zero and italics if they do. In model names, “int” represents the model intercept. We modeled region with eastern Pennsylvania = 0 and central Pennsylvania = 1. For each model set, we show either all models or the top five models and the null. See Appendix 4 for a comprehensive presentation of life phase survival models.

### 3.3 Factors driving nest survival

**FIGURE 2:**
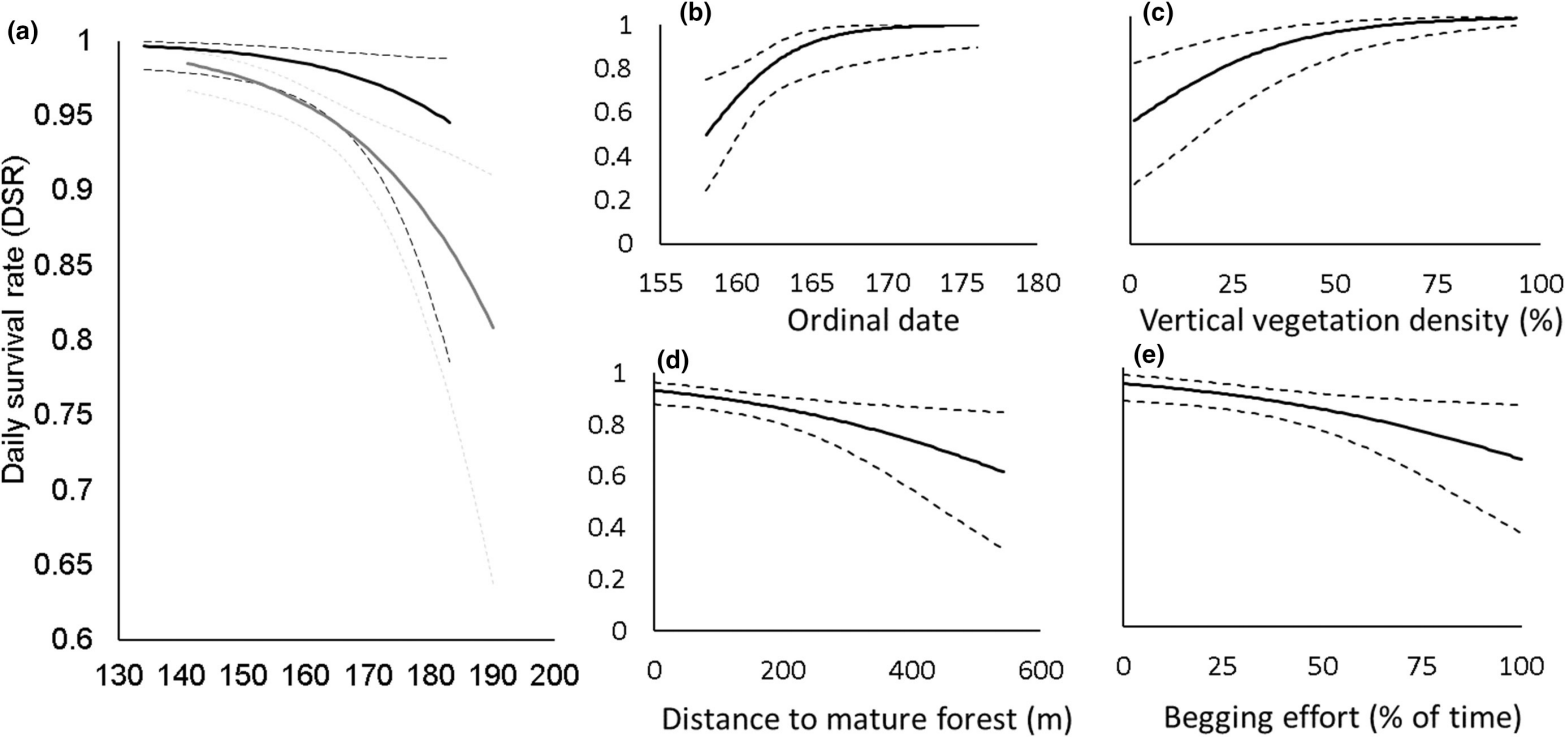
Model predictions for daily survival rate of golden‐winged warbler (*Vermivora chrysoptera*) nests in eastern Pennsylvania (solid black line; a) and central Pennsylvania (solid gray line; a). Additionally, we present modeled relationships for supported models explaining variation in fledgling survival (Known Fate, Program MARK) from eastern Pennsylvania (b) and central Pennsylvania (c–e). All solid lines represent model estimates while dashed lines represent 95% confidence intervals. Explanatory variables are only predicted across the observed ranges in each dataset. Predictions were made using the top‐ranked model in respective model sets (also see Tables 2 and 3).

As expected from our raw nest success data, nests in eastern Pennsylvania experienced higher survival rates (DSR = 0.97, 95% CI: 0.96–0.98) than those in central Pennsylvania (DSR = 0.95, 95% CI: 0.94–0.96). Nest survival declined as the breeding seasons progressed in both landscapes (Table 2; Appendix 5; Figure 2). Although models with habitat covariates ranked highly in both landscapes, models without habitat covariates (i.e., null models) were always competing and habitat term *β* 95% confidence intervals included zero, suggesting weak relationships with nest survival (Table 2).

**TABLE 2:** Models explaining survival of golden‐winged warbler (*Vermivora chrysoptera*) nest survival in eastern Pennsylvania and central Pennsylvania regions.

Model name	*k*	ΔAICc	*w*	Mod lik.	*β* coefficients
Eastern Pennsylvania					
Φ (int + ordinal + % woody [1 m])	3	0.00	0.06	1.00	*β*_1_: **−0.06**, *β*_2_: −*0.01*
Φ (int + ordinal + % *Rubus* [1 m])	3	0.08	0.05	0.96	*β*_1_: −**0.06**, *β*_2_: *0.02*
Φ (int + ordinal + sapling density [5 m])	3	0.17	0.05	0.92	*β*_1_: −**0.06**, *β*_2_: −*0.01*
Φ (int + ordinal)	2	0.39	0.05	0.82	*β*_1_: −**0.06**
Φ (int + ordinal + % leaf litter[1 m])	3	0.73	0.04	0.69	*β*_1_: −**0.06**, *β*_2_:*0.05*
Φ (int)	1	3.37	0.01	0.19	—
Central Pennsylvania					
Φ (int + ordinal + % forb [11.3 m])	3	0.00	0.09	1.00	*β*_1_: −**0.06**, *β*_2_: *0.01*
Φ (int + ordinal)	2	0.73	0.06	0.69	*β*_1_: −**0.05**
Φ (int + ordinal + basal area)	3	0.90	0.05	0.64	*β*_1_: −**0.06**, *β*_2_: *0.08*
Φ (int + ordinal + % *Rubus* [1 m])	3	0.99	0.05	0.61	*β*_1_: −**0.06**, *β*_2_: −*0.01*
Φ (int + ordinal + % bare ground [1 m])	3	1.30	0.04	0.52	*β*_1_: −**0.06**, *β*_2_: −*0.32*
Φ (int)	1	10.08	0.00	0.01	—

*Note*: Shown are the number of model parameters (*k*), Δ Akaike's Information Criterion adjusted for small sample size (AICc), AICc weight (*w*) and model likelihood (Mod lik.) for candidate nest survival models. We do not present *β* coefficients for nest survival models because ordinal date‐only models were competing for both sets (eastern Pennsylvania/central Pennsylvania). For each presented, model *β* coefficients are also shown where *β*_0_ (not shown) represents the intercept and *β*_1_ … *β*_k_ represents model covariate slopes. Model *β* coefficients are presented in bold if their *β* 95% confidence intervals that do not include zero and italics if they do. For each model set, present the top five models and the intercept‐only null. See Appendix 5 for a comprehensive presentation of nest survival models.

### 3.4 Factors driving fledgling survival

Fledgling survival varied with individual factors, microhabitat, and stand‐scale habitat factors (Table 3, Appendix 6). Fledgling survival in eastern Pennsylvania was a function of ordinal date (positive relationship) but vegetation covariates were not strongly associated with fledgling DSR (Figure 2, Table 3). Fledgling survival in central Pennsylvania was a function of percent begging effort (negative relationship), vertical vegetation density (positive relationship), and distance to mature forest (negative relationship; Figure 2, Table 3). Interestingly, although comparisons of body condition were not a primary objective of this study, differences in weight and behavior between the regions were stark; fledglings in eastern Pennsylvania were 10% heavier than those in central Pennsylvania, whether marked as nestlings (eastern Pennsylvania: 8.36 g, 95% CI: 8.22–8.50 vs. central Pennsylvania: 7.64 g, 95% CI: 7.38–7.89) or fledglings (eastern Pennsylvania: 9.02, 95% CI: 8.37–9.66 vs. central Pennsylvania: 7.80, 95% CI: 7.62–7.99). Additionally, eastern Pennsylvania fledglings begged for provisioning half as frequently (mean [days 1–5]: 13%, 95% CI: 11–16%) than central Pennsylvania fledglings (mean [days 1–5]: 26%, 95% CI: 23–29%).

**TABLE 3:** Models explaining survival of golden‐winged warbler (*Vermivora chrysoptera*) fledgling survival in eastern Pennsylvania and central Pennsylvania regions.

Model name	*k*	ΔAICc	*w*	Mod lik.	*β* coefficients
Eastern Pennsylvania – Individual factors					
Φ (int + age + fledge date)	3	0.00	0.94	1.00	*β*_1_: **−51.40**, *β*_2_: **0.35**
Φ (int)	1	30.91	0.00	0.00	—
Eastern Pennsylvania – Micro‐habitat					
Φ (Int + age + % unvegetated)	3	0.00	0.47	1.00	*β*_1_: **3.54**, *β*_2_: *0.08*
Φ (int)	1	26.07	0.00	0.00	—
Eastern Pennsylvania – Stand‐scale habitat					
Φ (int + age + DTN shrub‐wetland)	3	0.00	0.23	1.00	*β*_1_: **5.76**, *β*_2_: *−0.01*
Φ (int + age + DTN swamp)	3	0.20	0.21	0.90	*β*_1_: **5.31**, *β*_2_: *−0.01*
Φ (int + age + % mature forest)	3	0.39	0.19	0.82	*β*_1_: **4.09**, *β*_2_: *0.03*
Φ (Int + age)	2	1.31	0.12	0.52	*β*_1_: **1.36**
Φ (int + age + DTN mature forest)	3	1.77	0.09	0.41	*β*_1_: **5.01**, *β*_2_: *−0.01*
Φ (int)	1	24.24	0.00	0.00	—
Central Pennsylvania – Individual factors					
Φ (int + begging effort)	2	0.00	0.42	1.00	*β*_1_: **0.36**
Φ (int + age + begging effort)	3	1.46	0.20	0.48	*β*_1_: **2.71**, *β*_2_: **−0.42**
Φ (int)	1	3.15	0.09	0.21	—
Central Pennsylvania – Micro‐habitat					
Φ (int + vertical veg. density)	2	0.00	0.55	1.00	*β*_1_: **0.04**
Φ (int + age + vertical veg. density)	3	0.43	0.45	0.81	*β*_1_: **0.61**, *β*_2_: **0.05**
Φ (int)	1	21.76	0.00	0.00	—
Central Pennsylvania – Stand‐scale habitat					
Φ (int + DTN mature forest)	2	0.00	0.44	1.00	*β*_1_: **−0.01**
Φ (int + age + DTN mature forest)	3	1.83	0.18	0.4	*β*_1_: **2.56**, *β*_2_: **−0.01**
Φ (int)	1	3.82	0.06	0.15	—

*Note*: Shown are the number of model parameters (*k*), Δ Akaike's Information Criterion adjusted for small sample size (AICc), AICc weight (*w*) and model likelihood (Mod lik.) for candidate nest survival models. For competing models (ΔAICc <2), model *β* coefficients are also shown where *β*_0_ (not shown) represents the intercept and *β*_1_ … *β*_k_ represents model covariate slopes. *β* coefficients are presented in bold if their *β* 95% confidence intervals that do not include zero and italics if they do. In model names, “int” represents the model intercept. Below we present competing models and the intercept‐only null. See Appendix 6 for a comprehensive presentation of fledgling survival models.

## 4 DISCUSSION

Understanding the drivers of variation in breeding productivity rates can be critical to the conservation of imperiled species (Bookhout, 1994; Primack, 2006). Contrary to our expectations, our analyses demonstrate that substantial differences in breeding productivity (3.07 juveniles/pair/year in eastern Pennsylvania vs 1.08 in central Pennsylvania) appear to be driven by variable survival rates of nestlings and young fledglings—an approximately 15‐day window in the species' lifecycle. Had we examined only habitat conditions' influence on nest survival, we would have revealed no manageable habitat features for which conservationists should target in future habitat management (e.g., vertical vegetation density, Table 1; McNeil et al., 2017). In contrast, a fledgling‐only view of productivity would have failed to capture the important contribution of nest survival to region‐specific breeding productivity (Figure 1). Simultaneous consideration of both components of productivity provides a more nuanced insight into the habitat needs of species like the golden‐winged warbler for which nest‐ and fledgling survival vary interdependently (Rush & Stutchbury, 2008; Schmidt et al., 2008). For example, although nesting habitat guidelines suggest specific targets for management to maximize nest survival, our findings imply a need for dense patches of vegetation (below 2 m) within and around nesting sites to enhance fledgling survival (Table 1).

Although we identified several habitat features that influenced fledgling survival, much of the variation that drove differential breeding output between the two regions could only be attributed to low nestling‐ and young fledgling survival in central Pennsylvania (Figure 1). While we primarily focused on structural habitat characteristics as survival covariates here, we pose a new hypothesis that would explain variation in this system: food limitation in central Pennsylvania. This idea is supported by the marked differences in mass we observed between fledglings of the two regions (central Pennsylvania: 7.64 g vs. eastern Pennsylvania: 8.36 g). Interestingly, the fledgling mass in eastern Pennsylvania was comparable to that reported in Minnesota, where the population size is relatively stable (8.6 g; Streby et al., 2016). In addition to strong mass differences, contrasting begging behavior (central Pennsylvania: 26% vs. eastern Pennsylvania: 13%) may also lend support for this hypothesis. Begging, which reflects hunger (Hinde & Godfray, 2011), is considered a relatively risky behavior (Godfray & Johnstone, 2000; Trivers, 1985), perhaps even more so during the first few days post‐fledging (Jones et al., 2020; Naef‐Daenzer & Grüebler, 2016). Perhaps not surprisingly then, begging was negatively related to fledgling survival, though only in central Pennsylvania (Table 3; Figure 2). Finally, a food‐limitation hypothesis is also supported by our finding that egg‐stage nest DSR was equal between the landscapes while nestling‐stage nest DSR was lower in central Pennsylvania (Figure 1). Future work that explicitly tests this hypothesis would be valuable, perhaps by quantifying prey availability (especially leaf‐rolling caterpillars [e.g., *Pandemis* spp., *Episimus* spp., etc.]; Streby et al., 2014) or measuring indicators of food stress (e.g., blood chemistry; Jenni‐Eiermann et al., 2008).

Although both regions we studied were characterized by comparable habitat conditions (within nesting habitat and adjacent post‐fledging habitats; Fiss et al., 2020, 2021) and focal sites were created using identical best management practices (Bakermans et al., 2011; Roth et al., 2019), habitat effects were only detected in central Pennsylvania (but not eastern Pennsylvania fledglings or nests from either region; Table 3). Survival of young (≤6 days post‐fledging) fledglings was related to both microhabitat and stand‐scale factors in central Pennsylvania but only individual factors (i.e., ordinal date; Schmidt et al., 2008) in eastern Pennsylvania. Although we pose a hypothesis here related to prey availability that explains much of the variation we observe in the system, other ecological phenomena may also contribute to vital rate differences between central‐ and eastern Pennsylvania, such as potential differences in predator communities. Another factor worth bearing in mind is the potential effect of weather on warbler demography. While we assume that weather‐driven variation was random and we believe that weather conditions were comparable between our focal regions (Appendix 1), a study that examined all aspects of demography in both regions, simultaneously, and over more than 2 years would greatly reduce any such bias. With that in mind, our finding that years differed within each region (Appendix 1), yet we still detected no average “year” effect on nest/fledgling survival (Appendices 5 and 6) strongly suggests that annual weather patterns, in the context of our study, played a minor role in warbler reproductive demography. Collectively, our findings of landscape‐specific patterns underscore the importance of assessing survival across landscapes, even when a single habitat type/management prescription is studied (Confer et al., 2010; King et al., 2006; McNeil et al., 2018; Vitz & Rodewald, 2007, 2011). This is especially important considering our work is the first study to assess ecological influences on golden‐winged warbler post‐fledging survival in the Appalachian portion of the species' breeding range (Rohrbaugh et al., 2016; also see Lehman, 2017) where the populations have been declining for at least a half‐century (Sauer et al., 2020).

## 5 CONCLUSION

Conservation efforts for the golden‐winged warbler have gained momentum over the past decade (McNeil, Rodewald, Ruiz‐Gutierrez, et al., 2020) and these efforts have focused heavily on portions of the Appalachian sub‐population (Litvaitis et al., 2021). Although a strong conservation focus in this region is warranted (Rohrbaugh et al., 2016; Sauer et al., 2020), poor responses to breeding habitat creation in central Pennsylvania contrasting with those in eastern Pennsylvania have puzzled conservationists (McNeil, 2019; McNeil, Rodewald, Ruiz‐Gutierrez, et al., 2020). By incorporating both nest and fledgling survival data together, and by decomposing these metrics into finer components, we identified previously undescribed bottlenecks in local population‐specific breeding output in this species. While conservation plans for species like the golden‐winged warbler commonly consider the needs of nesting adults (Rosenberg et al., 2003; Roth et al., 2019; Wood et al., 2013), they only rarely consider the needs of fledglings (Cox et al., 2014), which is one of the most limiting portions of the songbird annual cycle (Jones et al., 2020; Naef‐Daenzer & Grüebler, 2016). For instance, golden‐winged warblers nest in early successional communities but commonly use more diverse cover (e.g., mature forest, pole timber, etc.) after fledging (Fiss et al., 2020, 2021) and other forest birds exhibit similar habitat shifts (McNeil, 2019). Our results add to the growing body of evidence that best management plans for forest bird species (e.g., Cerulean Warbler, *Setophaga cerulea*; Wood Thrush, *Hylocichla mustelina*) should explicitly consider post‐fledging habitat needs to maximize conservation efficacy (Rosenberg et al., 2003; Wood et al., 2013). In the case of the golden‐winged warbler, managing forests in a “dynamic” fashion whereby a variety of forest age classes are created within close proximity is likely to ensure that the post‐fledging habitat is always within close proximity to the nesting habitat (Fiss et al., 2020, 2021; McNeil et al., 2023). While our study provides new insights into demographic contributors to songbird productivity, further research is still needed to understand how stage‐specific survival varies with habitat and landscape attributes. In particular, additional research on factors influencing independent juvenile survival during the post‐breeding period is needed, as this life stage remains a largely undescribed component of the lifecycle of the golden‐winged warbler (Rohrbaugh et al., 2016, though see Streby et al., 2015) and migratory songbirds in general (Greenberg & Marra, 2005).

## AUTHOR CONTRIBUTIONS

**Darin J. McNeil:** Conceptualization (equal); data curation (equal); formal analysis (equal); investigation (equal); methodology (equal); validation (equal); visualization (equal); writing – original draft (equal); writing – review and editing (equal). **Amanda D. Rodewald:** Conceptualization (equal); funding acquisition (equal); investigation (equal); methodology (equal); project administration (equal); resources (equal); supervision (equal); validation (equal); writing – original draft (equal); writing – review and editing (equal). **Viviana Ruiz‐Gutierrez:** Conceptualization (equal); data curation (equal); formal analysis (equal); methodology (equal); validation (equal); writing – review and editing (equal). **Cameron J. Fiss:** Conceptualization (equal); data curation (equal); investigation (equal); methodology (equal); validation (equal); writing – review and editing (equal). **Jeffery L. Larkin:** Conceptualization (equal); funding acquisition (equal); funding acquisition (equal); investigation (equal); investigation (equal); methodology (equal); methodology (equal); project administration (equal); project administration (equal); resources (equal); resources (equal); supervision (equal); supervision (equal); validation (equal); validation (equal); writing – original draft (equal); writing – original draft (equal); writing – review and editing (equal); writing – review and editing (equal).

## CONFLICT OF INTEREST STATEMENT

The authors have no competing interests.

## Data Availability

All MARK input files (which contain the data), README file, and MARK. DBF/.FPT files (which contain the analyses) can be accessed via Dryad at this link: https://datadryad.org/stash/share/MYDfIhUK1KkUO1kXS1HRJOfMt0qGgT9mMkBZTe6NTrI.

## APPENDIX 1

### 1.1

Periods of drought as defined by the National Drought Mitigation Center from May through July 2014–17 in eastern Pennsylvania (black) and central Pennsylvania (red). Drought index shown (%) drought is the mean for Pike + Monroe Counties (eastern Pennsylvania) and Center + Clinton Counties (central Pennsylvania).

## APPENDIX 2

### 2.1

Golden‐winged Warblers can be aged reliably to the day during the nestling stage. Shown is the progression of one Golden‐winged Warbler nest from the day of hatching to the moment right before fledging. Note differences in body size, coverage/location of pinfeathers, and advance of juvenal plumage development.

## APPENDIX 3

### 3.1

Ordinal dates of nest initiation for nests in eastern Pennsylvania (a) and central Pennsylvania (b), with the mean nest initiation date indicated by an arrow. Gray bars represent known second/third nesting attempts based on pairs with banded adults.

## APPENDIX 4

### 4.1

Models explaining survival of golden‐winged warbler (*Vermivora chrysoptera*) life phases in eastern Pennsylvania and central Pennsylvania regions. Shown are the number of model parameters (*k*), Δ Akaike's Information Criterion adjusted for small sample size (AICc), AICc weight (*w*) and model likelihood (Mod lik.) for candidate nest survival models. For competing models (ΔAICc <2); above the dashed line, all model *β* coefficients are shown where *β*_0_ (not shown) represents the intercept and *β*_1_ … *β*_k_ represents model covariate slopes. *β* coefficients are presented in bold if their *β* 95% confidence intervals that do not include zero and italics if they do. In model names, “int” represents the model intercept. We modeled region with eastern Pennsylvania = 0 and central Pennsylvania = 1. This information is presented, in part, in Table 1 of the main manuscript whereas this table comprehensive in terms of models run.

**Table:**

Model name	*k*	ΔAICc	*w*	Mod lik.	*β* coefficients
Egg‐phase survival					
Φ (int)	1	0.00	0.59	1.00	—
Φ (int + region)	2	0.76	0.41	0.68	*β*_1_: *−0.35*
Nestling‐phase survival					
Φ (int + region)	2	0.00	0.99	1.00	*β*_1_: **−1.23**
Φ (int)	1	9.66	0.01	0.01	—
Young (1–5 days Fledgling‐phase survival)					
Φ (int + d_2–5_ + region_1_ + region_2–5_)	4	0.00	0.51	1.00	*β*_1_: *0.81*, *β*_2_: **4.52**, *β*_3_: **−2.50**
Φ (int + d_2–5_ + region_2–5_)	3	0.14	0.48	0.93	*β*_1_: **4.52**, *β*_2_: **−2.50**
Φ (int + d_3–5_ + region_3–5_)	3	11.06	0.00	0.00	—
Φ (int + d_3–5_ + region_1–2_ + region_3–5_)	4	13.00	0.00	0.00	—
Φ (int + age + region)	3	17.19	0.00	0.00	—
Φ (int + d_2–5_ + region_1_)	3	17.69	0.00	0.00	—
Φ (int + d_2–5_)	2	17.83	0.00	0.00	—
Φ (int + d_3–5_)	2	19.17	0.00	0.00	—
Φ (int + age)	2	19.86	0.00	0.00	—
Φ (int + d_4–5_ + region_4–5_)	3	20.36	0.00	0.00	—
Φ (int + d_4–5_ + region_1–3_ + region_4–5_)	4	21.03	0.00	0.00	—
Φ (int + region)	2	21.05	0.00	0.00	—
Φ (int + d_3–5_ + region_1–2_)	3	21.10	0.00	0.00	—
Φ (int + age^2^ + region)	3	22.83	0.00	0.00	—
Φ (int + d_4‐5_)	2	23.61	0.00	0.00	—
Φ (int)	1	23.86	0.00	0.00	—
Φ (int + d_4–5_ + region_1–3_)	3	24.28	0.00	0.00	—
Φ (int + d_5_ + region_1–4_)	3	24.31	0.00	0.00	—
Φ (int + d_5_ + region_1–4_ + region_5_)	4	24.79	0.00	0.00	—
Φ (int + age^2^)	2	25.53	0.00	0.00	—
Φ (int + d_5_)	2	25.65	0.00	0.00	—
Φ (int + d_5_ + region_5_)	3	26.13	0.00	0.00	—
Old (6–30 days Fledgling‐phase survival)					
Φ (int + region)	2	0.00	0.93	1.00	—
Φ (int)	1	5.27	0.07	0.07	—

## APPENDIX 5

### 5.1

Models explaining survival of golden‐winged warbler (*Vermivora chrysoptera*) nest survival in eastern Pennsylvania and central Pennsylvania regions. Shown are the number of model parameters (*k*), Δ Akaike's Information Criterion adjusted for small sample size (AICc), AICc weight (*w*) and model likelihood (Mod lik.) for candidate nest survival models. We do not present *β* coefficients for nest survival models because ordinal date‐only models were competing for both sets (eastern Pennsylvania/ central Pennsylvania). *β* coefficients on ordinal date were always negative and their 95% confidence intervals did not overlap zero. The models presented here are also presented, in part, in Table 2 of the main manuscript whereas this table comprehensive in terms of models run.

**Table:**

Model name	*k*	ΔAICc	*w*	Mod lik.
Eastern Pennsylvania				
Φ (int + ordinal + % woody [1 m])	3	0.00	0.06	1.00
Φ (int + ordinal + % *Rubus* [1 m])	3	0.08	0.05	0.96
Φ (int + ordinal + sapling density [5 m])	3	0.17	0.05	0.92
Φ (int + ordinal)	2	0.39	0.05	0.82
Φ (int + ordinal + % leaf litter[1 m])	3	0.73	0.04	0.69
Φ (int + ordinal + basal area)	3	0.97	0.03	0.62
Φ (int + ordinal + >2 m shrub density [5 m])	3	1.19	0.03	0.55
Φ (int + ordinal + sapling height [11.3 m])	3	1.31	0.03	0.52
Φ (int + ordinal + # snags [11.3 m])	3	1.38	0.03	0.5
Φ (int + ordinal + ordinal^2^ + % *Rubus* [1 m])	4	1.62	0.03	0.45
Φ (int + ordinal + % forb [1 m])	3	1.64	0.02	0.44
Φ (int + ordinal + % vine [11.3 m])	3	1.68	0.02	0.43
Φ (int + ordinal + % vine [1 m])	3	1.79	0.02	0.41
Φ (int + ordinal + ordinal^2^ + % woody [1 m])	4	1.80	0.02	0.41
Φ (int + ordinal + year)	3	1.84	0.02	0.40
Φ (int + ordinal + ordinal^2^ + sapling density [5 m])	4	1.95	0.02	0.38
Φ (int + ordinal + % Rubus [11.3 m])	3	1.99	0.02	0.37
Φ (int + ordinal + ordinal^2^)	3	1.99	0.02	0.37
Φ (int + ordinal + ordinal^2^ + % leaf litter [1 m])	4	2.28	0.02	0.32
Φ (int + ordinal + % grass [1 m])	3	2.28	0.02	0.32
Φ (int + ordinal + % bare ground [1 m])	3	2.29	0.02	0.32
Φ (int + ordinal +1–2 m shrub density [5 m])	3	2.35	0.02	0.31
Φ (int + ordinal + shrub height [11.3 m])	3	2.36	0.02	0.31
Φ (int + ordinal + % forb [11.3 m])	3	2.39	0.02	0.30
Φ (int + ordinal + % grass [11.3 m])	3	2.39	0.02	0.30
Φ (int + ordinal + ordinal^2^ + basal area)	4	2.57	0.02	0.28
Φ (int + % woody [1 m])	2	2.61	0.02	0.27
Φ (int + % leaf litter [1 m])	2	2.65	0.01	0.27
Φ (int + % *Rubus* [1 m])	2	2.67	0.01	0.26
Φ (int + ordinal + ordinal^2^ + >2 m shrub density [5 m])	4	2.85	0.01	0.24
Φ (int + ordinal + ordinal^2^ + # snags [11.3 m])	4	2.96	0.01	0.23
Φ (int + sapling density [5 m])	2	2.96	0.01	0.23
Φ (int + ordinal + ordinal^2^ + sapling height [11.3 m])	4	3.00	0.01	0.22
Φ (int + basal area)	2	3.22	0.01	0.20
Φ (int + ordinal + ordinal^2^ + % vine [11.3 m])	4	3.26	0.01	0.20
Φ (int)	1	3.37	0.01	0.19
Φ (int + ordinal + ordinal^2^ + % forb [1 m])	4	3.41	0.01	0.18
Φ (int + ordinal + ordinal^2^ + % vine [1 m])	4	3.42	0.01	0.18
Φ (int + >2 m shrub density [5 m])	2	3.45	0.01	0.18
Φ (int + ordinal + ordinal^2^ + year)	4	3.49	0.01	0.17
Φ (int + ordinal + ordinal^2^ + % *Rubus* [11.3 m])	4	3.57	0.01	0.17
Φ (int + ordinal + ordinal^2^ + % grass [1 m])	4	3.88	0.01	0.14
Φ (int + ordinal + ordinal^2^ + % bare ground [1 m])	4	3.92	0.01	0.14
Φ (int + ordinal + ordinal^2^ + shrub height [11.3 m])	4	3.97	0.01	0.14
Φ (int + ordinal + ordinal^2^ + 1–2 m shrub density [5 m])	4	3.98	0.01	0.14
Φ (int + ordinal + ordinal^2^ + % forb [11.3 m])	4	3.99	0.01	0.14
Φ (int + ordinal + ordinal^2^ + % grass [11.3 m])	4	4.01	0.01	0.13
Φ (int + year)	2	4.20	0.01	0.12
Φ (int + % vine [11.3 m])	2	4.39	0.01	0.11
Φ (int + % vine [1 m])	2	4.49	0.01	0.11
Φ (int + # snags [11.3 m])	2	4.60	0.01	0.10
Φ (int + % forb [1 m])	2	4.67	0.01	0.10
Φ (int + sapling height [11.3 m])	2	4.85	0.00	0.09
Φ (int + % bare [1 m])	2	5.23	0.00	0.07
Φ (int + % grass [1 m])	2	5.25	0.00	0.07
Φ (int + shrub height [11.3 m])	2	5.25	0.00	0.07
Φ (int + % *Rubus* [11.3 m])	2	5.25	0.00	0.07
Φ (int + % grass [11.3 m])	2	5.32	0.00	0.07
Φ (int + % forb [11.3 m])	2	5.32	0.00	0.07
Φ (int + 1–2 m shrub density [5 m])	2	5.37	0.00	0.07
Central Pennsylvania				
Φ (int + ordinal + % forb [11.3 m])	3	0.00	0.09	1.00
Φ (int + ordinal)	2	0.73	0.06	0.69
Φ (int + ordinal + basal area)	3	0.90	0.05	0.64
Φ (int + ordinal + % *Rubus* [1 m])	3	0.99	0.05	0.61
Φ (int + ordinal + % bare ground [1 m])	3	1.30	0.04	0.52
Φ (int + ordinal + % vine [1 m])	3	1.79	0.03	0.41
Φ (int + ordinal + % forb [1 m])	3	1.82	0.03	0.40
Φ (int + ordinal + ordinal^2^ + % forb [11.3 m])	4	1.86	0.03	0.39
Φ (int + ordinal + % *Rubus* [11.3 m])	3	2.13	0.03	0.34
Φ (int + ordinal + shrub height [11.3 m])	3	2.15	0.03	0.34
Φ (int + ordinal + % woody [1 m])	3	2.16	0.03	0.34
Φ (int + ordinal + ordinal^2^ + % *Rubus* [1 m])	4	2.27	0.03	0.32
Φ (int + ordinal + # snags [11.3 m])	3	2.31	0.03	0.31
Φ (int + ordinal + ordinal^2^)	3	2.37	0.03	0.31
Φ (int + ordinal +1–2 m shrub density [5 m])	3	2.42	0.03	0.30
Φ (int + ordinal + >2 m shrub density [5 m])	3	2.45	0.03	0.29
Φ (int + ordinal + sapling density [5 m])	3	2.52	0.02	0.28
Φ (int + ordinal + ordinal^2^ + basal area)	4	2.53	0.02	0.28
Φ (int + ordinal + % grass [11.3 m])	3	2.53	0.02	0.28
Φ (int + ordinal + sapling height [11.3 m])	3	2.66	0.02	0.26
Φ (int + ordinal + % grass [1 m])	3	2.68	0.02	0.26
Φ (int + ordinal + year)	3	2.69	0.02	0.26
Φ (int + ordinal + % vine [11.3 m])	3	2.72	0.02	0.26
Φ (int + ordinal + % leaf litter[1 m])	3	2.73	0.02	0.26
Φ (int + ordinal + ordinal^2^ + % bare ground [1 m])	4	2.79	0.02	0.25
Φ (int + ordinal + ordinal^2^ + % vine [1 m])	4	3.43	0.02	0.18
Φ (int + ordinal + ordinal^2^ + % *Rubus* [11.3 m])	4	3.46	0.02	0.18
Φ (int + ordinal + ordinal^2^ + % forb [1 m])	4	3.49	0.01	0.17
Φ (int + ordinal + ordinal^2^ + shrub height [11.3 m])	4	3.79	0.01	0.15
Φ (int + ordinal + ordinal^2^ + % woody [1 m])	4	3.95	0.01	0.14
Φ (int + ordinal + ordinal^2^ + >2 m shrub density [5 m])	4	4.01	0.01	0.13
Φ (int + ordinal + ordinal^2^ + # snags [11.3 m])	4	4.05	0.01	0.13
Φ (int + ordinal + ordinal^2^ + 1–2 m shrub density [5 m])	4	4.06	0.01	0.13
Φ (int + ordinal + ordinal^2^ + % grass [11.3 m])	4	4.13	0.01	0.13
Φ (int + ordinal + ordinal^2^ + sapling density [5 m])	4	4.20	0.01	0.12
Φ (int + ordinal + ordinal^2^ + sapling height [11.3 m])	4	4.27	0.01	0.12
Φ (int + ordinal + ordinal^2^ + % grass [1 m])	4	4.31	0.01	0.12
Φ (int + ordinal + ordinal^2^ + year)	4	4.34	0.01	0.11
Φ (int + ordinal + ordinal^2^ + % leaf litter [1 m])	4	4.38	0.01	0.11
Φ (int + ordinal + ordinal^2^ + % vine [11.3 m])	4	4.38	0.01	0.11
Φ (int + basal area)	2	8.87	0.00	0.01
Φ (int + % forb [11.3 m])	2	9.33	0.00	0.01
Φ (int)	1	10.08	0.00	0.01
Φ (int + % forb [1 m])	2	11.02	0.00	0.00
Φ (int + % woody [1 m])	2	11.21	0.00	0.00
Φ (int + % bare [1 m])	2	11.42	0.00	0.00
Φ (int + % *Rubus* [1 m])	2	11.48	0.00	0.00
Φ (int + % *Rubus* [11.3 m])	2	11.53	0.00	0.00
Φ (int + >2 m shrub density [5 m])	2	11.56	0.00	0.00
Φ (int + shrub height [11.3 m])	2	11.60	0.00	0.00
Φ (int + % vine [11.3 m])	2	11.65	0.00	0.00
Φ (int + # snags [11.3 m])	2	11.81	0.00	0.00
Φ (int + % vine [1 m])	2	11.90	0.00	0.00
Φ (int + sapling density [5 m])	2	11.91	0.00	0.00
Φ (int + 1–2 m shrub density [5 m])	2	11.96	0.00	0.00
Φ (int + % grass [11.3 m])	2	12.03	0.00	0.00
Φ (int + year)	2	12.04	0.00	0.00
Φ (int + % leaf litter [1 m])	2	12.06	0.00	0.00
Φ (int + sapling height [11.3 m])	2	12.08	0.00	0.00
Φ (int + % grass [1 m])	2	12.09	0.00	0.00

## APPENDIX 6

### 6.1

Models explaining survival of golden‐winged warbler (*Vermivora chrysoptera*) fledgling survival in eastern Pennsylvania and central Pennsylvania regions. Shown are the number of model parameters (*k*), Δ Akaike's Information Criterion adjusted for small sample size (AICc), AICc weight (*w*) and model likelihood (Mod lik.) for candidate nest survival models. For competing models (ΔAICc <2), model *β* coefficients are also shown where *β*_0_ (not shown) represents the intercept and *β*_1_ … *β*_k_ represents model covariate slopes. *β* coefficients are presented in bold if their *β* 95% confidence intervals that do not include zero and italics if they do. In model names, “int” represents the model intercept. This information is presented, in part, in Table 3 of the main manuscript whereas this table comprehensive in terms of models run. In our stand‐scale analyses, “DTN” is shorthand for “distance‐to‐nearest.”

**Table:**

Model name	*k*	ΔAICc	*w*	Mod lik.	*β* coefficients
Eastern Pennsylvania – Individual factors					
Φ (int + age + fledge date)	3	0.00	0.94	1.00	*β*_1_: **−51.40**, *β*_2_: **0.35**
Φ (int + age)	2	7.98	0.02	0.02	—
Φ (int + age + distance moved)	3	8.43	0.01	0.01	—
Φ (int + age + mass)	3	8.87	0.01	0.01	—
Φ (int + age + begging)	3	9.93	0.01	0.01	—
Φ (int + age + year)	3	10.03	0.01	0.01	—
Φ (int + fledge date)	2	21.48	0.00	0.00	—
Φ (int + distance moved)	2	24.19	0.00	0.00	—
Φ (int + begging effort)	2	26.12	0.00	0.00	—
Φ (int)	1	30.91	0.00	0.00	—
Φ (int + mass)	2	31.62	0.00	0.00	—
Φ (int + year)	2	32.94	0.00	0.00	—
Eastern Pennsylvania – Micro‐habitat					
Φ (Int + age + % unvegetated)	3	0.00	0.47	1.00	*β*_1_: **3.54**, *β*_2_: *0.08*
Φ (int + age + vertical veg. density)	3	2.11	0.16	0.35	—
Φ (int + age)	2	3.14	0.10	0.21	—
Φ (int + age + % non‐herbaceous)	3	4.07	0.06	0.13	—
Φ (int + age + % herbaceous)	3	4.24	0.06	0.12	—
Φ (int + age + # snags)	3	4.37	0.05	0.11	—
Φ (int + age + basal area)	3	4.48	0.05	0.11	—
Φ (int + age + lateral veg. density)	3	4.69	0.05	0.10	—
Φ (int + % unvegetated)	2	20.82	0.00	0.00	—
Φ (int + vertical veg. density)	2	23.85	0.00	0.00	—
Φ (int)	1	26.07	0.00	0.00	—
Φ (int + # snags)	2	26.43	0.00	0.00	—
Φ (int + % non‐herbaceous)	2	26.53	0.00	0.00	—
Φ (int + % herbaceous cover)	2	26.69	0.00	0.00	—
Φ (int + lateral veg. density)	2	26.99	0.00	0.00	—
Φ (int + basal area)	2	27.29	0.00	0.00	—
Eastern Pennsylvania – Stand‐scale habitat					
Φ (int + age + DTN shrub‐wetland)	3	0.00	0.23	1.00	*β*_1_: **5.76**, *β*_2_: *−0.01*
Φ (int + age + DTN swamp)	3	0.20	0.21	0.90	*β*_1_: **5.31**, *β*_2_: *−0.01*
Φ (int + age + % mature forest)	3	0.39	0.19	0.82	*β*_1_: **4.09**, *β*_2_: *0.03*
Φ (Int + age)	2	1.31	0.12	0.52	*β*_1_: **1.36**
Φ (int + age + DTN mature forest)	3	1.77	0.09	0.41	*β*_1_: **5.01**, *β*_2_: *−0.01*
Φ (int + age + DTN pole sapling stand)	3	2.77	0.06	0.25	—
Φ (int + age + percent swamp)	3	2.80	0.06	0.25	—
Φ (int + age + DTN pole sapling stand)	3	3.02	0.05	0.22	—
Φ (int + DTN swamp)	2	22.16	0.00	0.00	—
Φ (int + DTN shrub‐wetland)	2	22.18	0.00	0.00	—
Φ (int + % mature forest)	2	23.51	0.00	0.00	—
Φ (int)	1	24.24	0.00	0.00	—
Φ (int + DTN mature forest)	2	25.16	0.00	0.00	—
Φ (int + percent swamp)	2	25.62	0.00	0.00	—
Φ (int + DTN pole sapling stand)	2	25.62	0.00	0.00	—
Φ (int + DTN thinned stand)	2	25.96	0.00	0.00	—
Central Pennsylvania – Individual factors					
Φ (int + begging effort)	2	0.00	0.42	1.00	*β*_1_: **0.36**
Φ (int + age + begging effort)	3	1.46	0.20	0.48	*β*_1_: **2.71**, *β*_2_: **−0.42**
Φ (int)	1	3.15	0.09	0.21	—
Φ (int + distance moved)	2	4.11	0.05	0.13	—
Φ (int + fledge date)	2	4.47	0.05	0.11	—
Φ (int + mass)	2	4.60	0.04	0.10	—
Φ (int + year)	2	4.98	0.03	0.08	—
Φ (int + age)	2	5.04	0.03	0.08	—
Φ (int + age + distance moved)	3	5.22	0.03	0.07	—
Φ (int + age + fledge date)	3	6.39	0.02	0.04	—
Φ (int + age + mass)	3	6.49	0.02	0.04	—
Φ (int + age + year)	3	6.90	0.01	0.03	—
Central Pennsylvania – Micro‐habitat					
Φ (int + vertical veg. density)	2	0.00	0.55	1.00	*β*_1_: **0.04**
Φ (int + age + vertical veg. density)	3	0.43	0.45	0.81	*β*_1_: **0.61**, *β*_2_: **0.05**
Φ (int + lateral veg. density)	2	13.05	0.00	0.00	—
Φ (int + age + lateral veg. density)	3	14.63	0.00	0.00	—
Φ (int + % unvegetated cover)	2	17.39	0.00	0.00	—
Φ (int + age + % unvegetated cover)	3	19.20	0.00	0.00	—
Φ (int + % herbaceous cover)	2	19.60	0.00	0.00	—
Φ (int + age + % herbaceous cover)	3	21.42	0.00	0.00	—
Φ (int)	1	21.76	0.00	0.00	—
Φ (int + % non‐herbaceous cover)	2	22.08	0.00	0.00	—
Φ (int + basal area)	2	23.44	0.00	0.00	—
Φ (int + # snags)	2	23.58	0.00	0.00	—
Φ (int + age)	2	23.65	0.00	0.00	—
Φ (int + age + % non‐herbaceous cover)	3	23.94	0.00	0.00	—
Φ (int + age + basal area)	3	25.33	0.00	0.00	—
Φ (int + age + # snags)	3	25.49	0.00	0.00	—
Central Pennsylvania – Stand‐scale habitat					
Φ (int + DTN mature forest)	2	0.00	0.44	1.00	*β*_1_: **−0.01**
Φ (int + age + DTN mature forest)	3	1.83	0.18	0.4	*β*_1_: **2.56**, *β*_2_: **−0.01**
Φ (int)	1	3.82	0.06	0.15	—
Φ (int + DTN thinned stand)	2	4.15	0.05	0.13	—
Φ (int + DTN upland shrubland)	2	4.25	0.05	0.12	—
Φ (int + DTN stem exclusion stand)	2	4.67	0.04	0.10	—
Φ (int + % mature forest)	2	5.29	0.03	0.07	—
Φ (int + age)	2	5.71	0.03	0.06	—
Φ (int + age + DTN thinned stand)	3	6.06	0.03	0.05	—
Φ (int + age + DTN upland shrubland)	3	6.18	0.02	0.05	—
Φ (int + age + DTN pole sapling stand)	3	6.57	0.02	0.04	—
Φ (int + age + % mature forest)	3	7.20	0.02	0.03	—
